# Correlation between spin structure oscillations and domain wall velocities

**DOI:** 10.1038/ncomms3328

**Published:** 2013-08-27

**Authors:** André Bisig, Martin Stärk, Mohamad-Assaad Mawass, Christoforos Moutafis, Jan Rhensius, Jakoba Heidler, Felix Büttner, Matthias Noske, Markus Weigand, Stefan Eisebitt, Tolek Tyliszczak, Bartel Van Waeyenberge, Hermann Stoll, Gisela Schütz, Mathias Kläui

**Affiliations:** 1 Department of Physics, University of Konstanz, Universitätsstrasse 10, 78457 Konstanz, Germany; 2 Max Planck Institute for Intelligent Systems, Heisenbergstrasse 3, 70569 Stuttgart, Germany; 3 SwissFEL, Paul Scherrer Institute, 5232 Villigen, Switzerland and Institute of Condensed Matter Physics, École Polytechnique Fédérale de Lausanne, 1015 Lausanne, Switzerland; 4 Institut für Physik, Johannes Gutenberg Universität Mainz, 55128 Mainz, Germany; 5 Swiss Light Source, Paul Scherrer Institute, 5232 Villigen, Switzerland; 6 Laboratory for Micro- and Nanotechnology, Paul Scherrer Institute, 5232 Villigen, Switzerland; 7 Institut für Optik und Atomare Physik, Technische Universität Berlin, 10623 Berlin, Germany; 8 Helmholtz-Zentrum Berlin für Materialien und Energie GmbH, Hahn-Meitner-Platz 1, 14109 Berlin, Germany; 9 Advanced Light Source, LBNL, Berkeley, California 94720, USA; 10 Department of Solid State Sciences, Ghent University, Krijgslaan 281 S1, 9000 Ghent, Belgium

## Abstract

Magnetic sensing and logic devices based on the motion of magnetic domain walls rely on the precise and deterministic control of the position and the velocity of individual magnetic domain walls in curved nanowires. Varying domain wall velocities have been predicted to result from intrinsic effects such as oscillating domain wall spin structure transformations and extrinsic pinning due to imperfections. Here we use direct dynamic imaging of the nanoscale spin structure that allows us for the first time to directly check these predictions. We find a new regime of oscillating domain wall motion even below the Walker breakdown correlated with periodic spin structure changes. We show that the extrinsic pinning from imperfections in the nanowire only affects slow domain walls and we identify the magnetostatic energy, which scales with the domain wall velocity, as the energy reservoir for the domain wall to overcome the local pinning potential landscape.

## Abstract (web summary)

A prerequisite for using domain walls in logic or sensing devices is a thorough knowledge of the properties and precise control. Here the authors monitor the domain wall motion in curved nanowires by stroboscopic imaging and find a regime of oscillating velocity and spin structure below the Walker breakdown.

## Graphical abstract

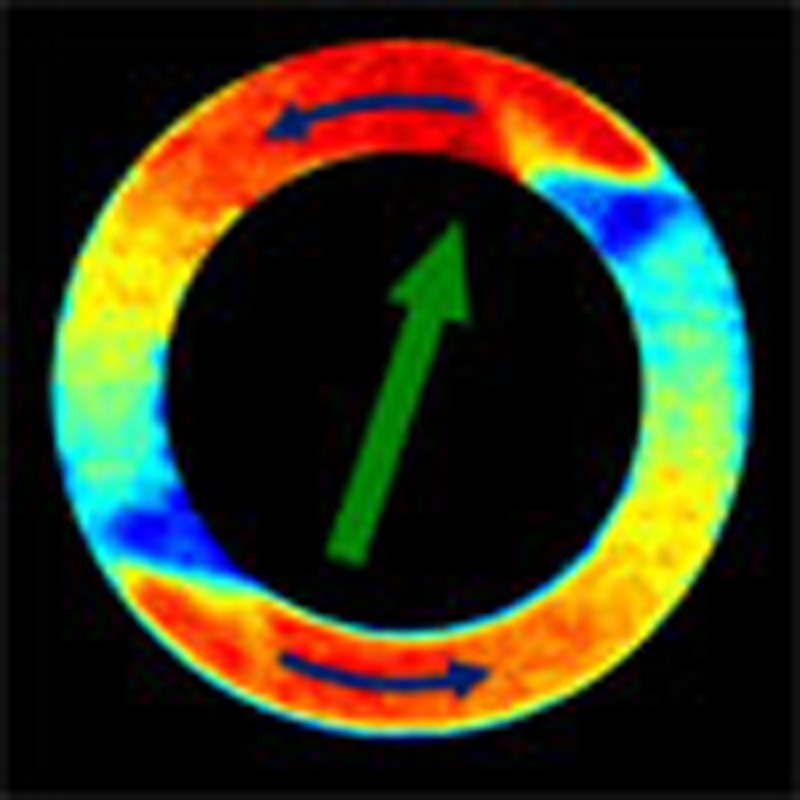

The key for the precise control of the position and velocity of magnetic domain walls in nanowires is a thorough understanding of the mechanisms that lead to variations in the domain wall velocity. Being independent of the driving mechanism, domain wall motion in ferromagnetic wires above the Walker breakdown^1^ exhibits periodic changes of the domain wall spin structure (precessional motion), leading to an intrinsically oscillating domain wall velocity^2^. On the other hand, imperfections in the nanowire, such as edge and surface roughness, give rise to a position dependence of the domain wall energy, resulting in velocity variations because of extrinsic pinning^3^. Although the coherent precession and the periodic spin structure changes of magnetic domain walls above the Walker breakdown have been electrically detected^4^, the imaging of the domain wall position and spin structure, necessary to understand the underlying physics, has been elusive. In addition, domain wall motion was shown to exhibit stochasticity because of extrinsic and intrinsic contributions to domain wall velocity variations^3^,^5^,^6^.

As a way to overcome the extrinsic pinning, the use of the domain wall inertia has been suggested^7^,^8^. Magnetic domain walls exhibiting inertia and effective mass have been reported for trapped domain walls in a pinning potential^7^,^9^,^10^,^11^,^12^,^13^,^14^, and vortex domain walls travelling several micrometres under their own inertia have been observed statically^15^ and by transport measurements^8^. Furthermore, theoretical predications have been made and the first indications show that the kinetic pinning field of moving domain walls is reduced compared with the static pinning^16^,^17^,^18^. This indicates that moving domain walls can overcome a local pinning potential, but the detailed mechanism that leads to vortex domain wall inertia for long-distance displacement has not been determined. However, it is exactly the quantitative understanding of the intrinsic and extrinsic effects that leads to domain wall velocity variations below and above the Walker breakdown that is the key to operation of magnetic logic and sensing devices^19^,^20^, which are based on domain wall propagation in *curved* nanowires, as the latter is so far the only application of domain walls that has made it to the market (Novotechnik, http://www.novotechnik.com/rsm)^20^.

In this work, we report direct dynamic experimental visualization of the oscillating propagation of domain walls in ferromagnetic nanorings driven by rotating magnetic field bursts. The oscillations of the vortex domain wall velocity are consequences of the interplay between the rotating driving field and the periodically changing domain wall spin structure. These periodic spin structure oscillations occur below and above the Walker breakdown and present a general feature in application-relevant curved geometries^19^,^20^.

## Results

### Direct dynamic imaging of domain wall propagation

**Figure 1:**
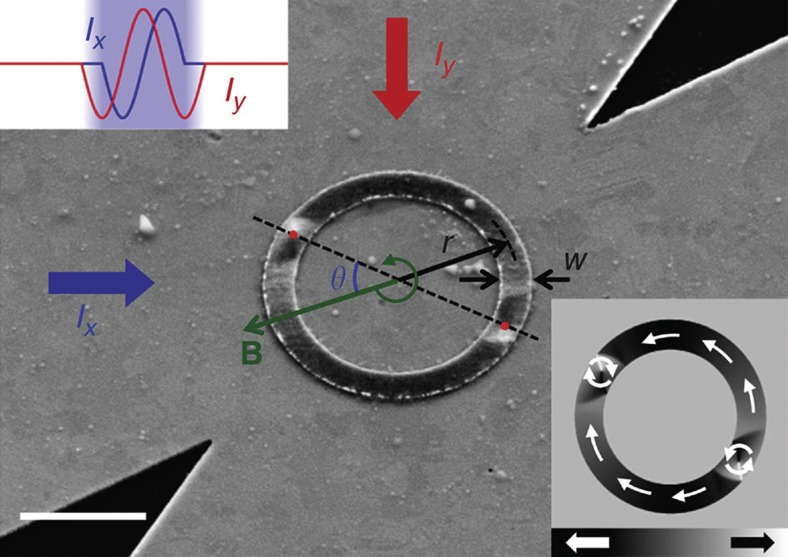
Experimental configuration. A scanning electron micrograph of a *w*=500 nm wide and 30 nm thick permalloy ring with radius *r*=2 μm covered by a 150nm thick copper-crossed stripline overlayed with a scanning transmission X-ray microscopy (STXM) image showing the in-plane magnetic contrast. The scale bar indicates a distance of 2 μm. After saturation, the ring is in the onion state where two vortex domain walls are formed, as illustrated by a micromagnetic simulation shown in the inset in the bottom right. The angle *θ* between the position of the domain walls and the applied field direction is indicated. The rotating magnetic field is generated by sinusoidal current bursts with a 90° phase shift injected in the horizontal and vertical crossed stripline.

Permalloy ring structures with 2–2.5 μm radius, 500–750 nm width and 30 nm thickness (see Methods)^21^,^22^ are, after saturation with a uniform magnetic field, in the ‘onion’ state, where two vortex domain walls are formed^23^,^24^,^25^, as shown in Fig. 1. When an external magnetic field **B** is applied, the Zeeman energy of the system depends on the angle *θ* between the field and the azimuthal position of the two vortex domain walls^26^ (the domain wall position is defined by the position of the vortex core). The total energy is minimal when the domain walls align with the applied field, providing a way to control their positions. By rotating the applied field, the energy potential landscape changes dynamically, leading to circular domain wall motion along the ring structure^26^. The domain wall velocity can be tuned by the field rotation frequency *f* independently from the field amplitude *B*, giving an extra degree of freedom to control the domain wall propagation compared with using the conventional straight wire geometry.

The dynamic domain wall propagation was imaged with sub-30 nm spatial resolution in a stroboscopic scheme, in which a snapshot of the moving domain walls is taken every 2 ns, employing time-resolved scanning transmission X-ray microscopy at the Advanced Light Source in Berkeley, CA, USA (beamline 11.0.2)^27^ and at the MAXYMUS endstation, Helmholtz Zentrum Berlin, BESSY II, Germany (see Methods). The domain walls are driven by counter-clockwise (CCW) rotating magnetic field burst pulses, in which the field amplitude first ramps up, pointing in the saturation direction of the onion state, followed by a complete CCW rotation of the in-plane magnetic field, and finally, the field amplitude is ramped down pointing in the original saturation direction of the onion state. These in-plane rotating magnetic field burst pulses are generated by sinusoidal current bursts injected into horizontal and vertical crossed striplines with a 90° phase shift^28^,^29^, as shown in Fig. 1. To achieve a high signal-to-noise ratio, the experiment is repeated at a repetition rate of 500 kHz and the transmission signal is recorded over >10^9^ subsequent pulsecycles and displacements of the domain walls. This allows us to determine not only the domain wall spin structure but also the reliability and reproducibility.

### Periodic domain wall spin structure oscillations

**Figure 2:**
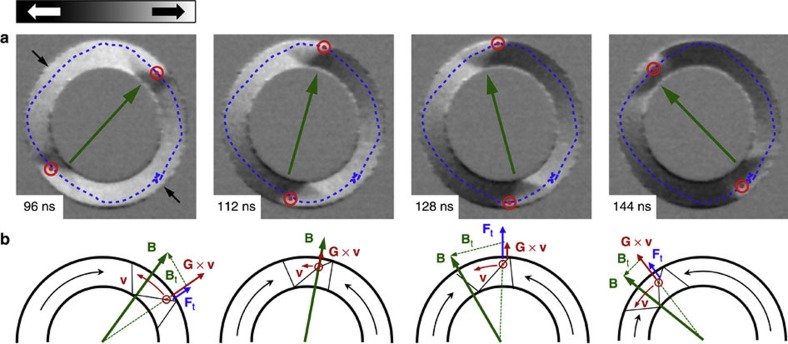
Direct imaging of periodic spin structure oscillations. (**a**) Time-resolved STXM snapshots of moving domain walls at *t*=96, 122, 128 and 144 ns during a 5MHz rotational field burst pulse are shown. The vortex core polarity and position (red) and the rotating field direction (green) are indicated by arrows. The dotted line (blue) shows the vortexcore trajectory of the vortex domain wall with clockwise chirality (*c*=−1). The rotating field pulse burst and the domain wall motion starts and stops at 135°, indicated by the the black arrows. (**b**) Schematic illustration of the relevant forces that act on the vortex domain wall spin-structure during propagation, corresponding to the images on top. The gyroforce **G** × **v** (red) and the force **F**_t_ (blue) from the tangential field component **B**_t_ are both acting radially on the vortex core.

Figure 2a shows four snapshots of two vortex domain walls in motion driven by CCW magnetic field burst pulses with a rotation frequency of *f*=5 MHz and field strength of *B*=6.8 mT in a ring structure (750 nm width, *r*=2 μm mean radius). The vortex core polarity of both vortex domain walls is positive (*p*=+1), as determined from the observed CCW vortex core gyration. The vortex core trajectory of the top domain wall (with clockwise chirality) is shown by the dotted blue line. The vortex domain wall spin structure and, in particular, the radial vortex core position are periodically oscillating at a frequency that is approximately four times faster than the field rotation frequency. The full movie of a complete field rotation can be found as Supplementary Movie 1.

**Figure 3:**
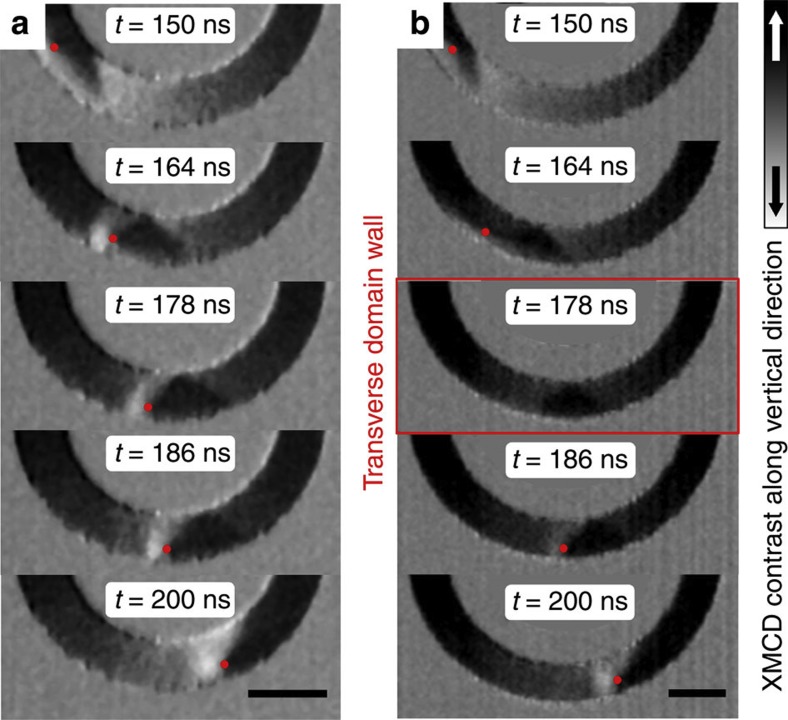
Domain wall transformation above the Walker breakdown. A series of time-resolved STXM snapshots at times *t*=150, 164, 178, 186 and 200 ns during a 5 MHz rotational field burst pulse with an amplitude of *B*=6.8 mT. The rings are 750 nm wide, 30 nm thick and have a radius of (**a**) *r*=2 μm and (**b**) *r*=2.5 μm. The scale bar indicates a distance of 1 μm. (**a**) Between *t*=164 and 186 ns, the vortex core with positive polarity *p*=+1 is pushed towards the outer edge of the ring structure. However, no domain wall transformation to the transverse wall spin structure occurs, and therefore, the domain wall propagates below the Walker breakdown. (**b**) At the same field-rotation frequency, but in a ring structure with larger radius, the vortex core is expelled at *t*=178 ns, such that the vortex domain wall transforms into a transverse domain wall. The vortex core then renucleates within 8 ns at the outer edge of the ring with positive vortex core polarity.

We imaged the domain wall propagation below and above the Walker breakdown. Above the Walker breakdown, the vortex domain wall transforms into a transverse domain wall by the expulsion and renucleation of the vortex core during the periodic spin structure oscillations, which can only be observed through direct dynamic imaging. To show this directly, we measured the domain wall propagation in two different ring structures (750 nm wide and 30 nm thick) with radii of *r*=2 μm and *r*=2.5 μm, as shown in Fig. 3a,b, respectively. In both cases, the field rotation frequency is 5 MHz and the field amplitude is *B*=6.8 mT. In case (**a**), the vortex core is pushed towards the outer edge of the ring, but is never expelled and the domain wall stays always a vortex wall and therefore, the domain wall propagates below the Walker breakdown. However, in case (**b**), with a larger ring radius, the vortex core is expelled at the outer edge of the ring structure and the vortex domain wall transforms into a transverse domain wall (see *t*=178 ns in Fig. 3b). The vortex core then renucleates at the outer edge of the ring structure with the same vortexcore polarity. Hence, not only the vortex core polarity, but also the chirality of the vortex domain wall is conserved during the Walker transformation in curved nanowires with rotating fields, in contrast to domain wall propagation above the Walker breakdown in straight nanowires, in which both the chirality and the polarity of the annihilated and the nucleated vortex alternate periodically^2^.

**Figure 4:**
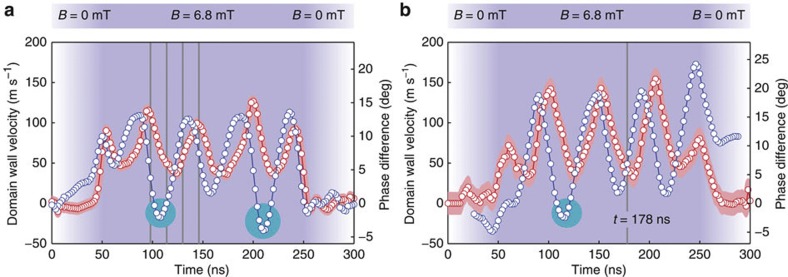
Oscillating domain wall velocity and phase difference. The domain wall velocity *v*_dw_ (red circles) and the phase difference *θ* (blue circles) are plotted as a function of time, observed (**a**) below the Walker breakdown and (**b**) above the Walker breakdown. The experimental error *δv*_dw_ is indicated by the red shade and the error of the phase difference is *δθ*=±2°. The amplitude *B* of the rotating magnetic field is indicated by the saturation of the background color. The phase difference is negative when the domain wall overshoots the rotating driving field, indicated by the circles in cyan. (**a**) The vertical lines (grey) indicate the points in time when the STXM snapshots in Fig. 2a were taken. (**b**) The vertical line (grey) shows the point in time when the vortex domain wall transforms into a transverse domain wall above the Walker breakdown, shown in Fig. 3b.

Simultaneously and with the same periodicity as the oscillation of the radial vortex core position, the domain wall velocity *v*_dw_ and the phase difference *θ* oscillate, as shown in Fig. 4a, b for the two cases (**a**) and (**b**), respectively. The domain wall velocity *v*_dw_ plotted is the magnitude of the angular velocity of the vortex core multiplied by the mean radius *r* of the ring structure; *v*_dw_ is positive for CCW domain wall motion and *vice versa*. The phase difference *θ* is the angle between the field **B** and the azimuthal vortex core position, as defined in Fig. 1. The average domain wall velocities are 67±2 ms^−1^ and 77±3 ms^−1^, and the maximum velocities are 126±15 ms^−1^ and 154±28 ms^−1^, respectively. Both the average and the maximum domain wall velocity are higher in case (**b**) for the ring structure with the larger radius and the resulting motion above the Walker breakdown. In Fig. 4, from 0 to 50 ns the field amplitude ramps up from *B*=0–6.8 mT; here the domain wall stays in its position along the field direction. In case (**a**), the phase difference increases because the vortex core is moving quasistatically, as its equilibrium position changes with increasing field amplitude and the domain wall velocity is small and negative. On the other hand, in case (**b**), the domain wall velocity is small and positive and the phase difference is negative because the chiralities of the vortex domain walls are reversed. For both cases, between 50 ns and 250 ns the domain wall velocity and the phase difference oscillate with a period of ~50 ns, corresponding to a frequency of 20 MHz, while the driving field rotates at a constant angular velocity and amplitude. In case (**b**), the vortex domain wall transforms into a transverse domain wall at 178 ns. Here the domain wall velocity is minimal, as expected for the Walker breakdown. After renucleation of the vortex core, the domain wall then accelerates while the vortex core moves towards its equilibrium position. At 110 and 210 ns (in Fig. 4a) and 120 ns (in Fig. 4b), the domain wall overshoots the driving field, resulting in a negative phase difference, which is a direct signature of the domain walls travelling under their own inertia^8^. At 250 ns, the field rotation stops abruptly and the vortex cores start to gyrate CCW around the equilibrium position while the field is being ramped down (250–300 ns).

**Figure 5:**
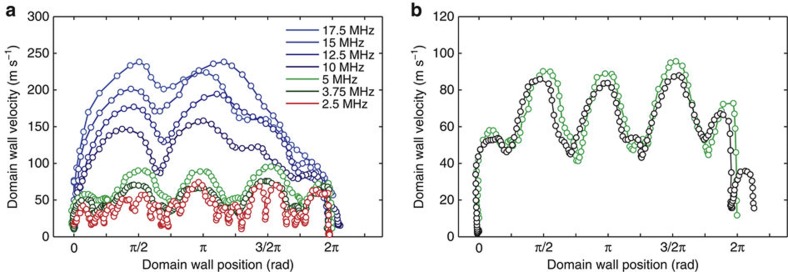
Oscillating domain wall velocity below and above the Walker breakdown. The domain wall velocity is plotted as a function of the domain wall position (**a**) for various field rotation frequencies of *f*=2.5–17.5 MHz and constant field amplitude *B*=6.8 mT, and (**b**) field amplides *B*=4.8 mT (black circles) and *B*=6.8 mT (green circles) and constant field rotation frequency *f*=5 MHz. The error of the domain wall velocities scales linearly with the field rotation frequency, from *δv*_dw_=±17 m s^−1^ at 2.5 MHz up to *δv*_dw_=±60 m s^−1^ at 17.5 MHz. (**a**) Pinning-dominated domain wall propagation (red), domain wall motion below (green) and above (blue) the Walker breakdown are indicated by the color. (**b**) The maximum domain wall velocity increases by a small but clearly visible amount for the higher field amplitude.

In straight nanowires, domain walls propagate below the Walker breakdown at a constant velocity. Only above the Walker breakdown, periodic transformations of the domain wall spin structure lead to oscillations of the domain wall velocity and these transformations significantly slow down the domain wall in average. Our observation in curved nanowires, however, shows domain wall velocity oscillations also below the Walker breakdown, even without any transformations of the domain wall spin structure from a vortex to a transverse wall. Moreover, we observe an oscillating domain wall velocity for all velocities, which means that the drastic changes at the Walker breakdown are absent in our geometry, further making it more suitable for applications where the critical behaviour at the Walker breakdown would lead to unreliable motion. This becomes particularly evident in our measurement of the domain wall velocity for various field rotation frequencies between 2.5–17.5 MHz, corresponding to average domain wall velocities of 31–220 ms^−1^, and field amplitudes of *B*=4.8 mT and *B*=6.8 mT. The ring structure is 500 nm wide, 30 nm thick and has a radius of 2 μm. In Fig. 5a, the domain wall velocities are plotted as functions of the domain wall position (azimuthal coordinate). Depending on the field rotation frequency, we identify three different domain wall propagation regimes. For *f*=2.5 MHz (red), we observe pinning-dominated, but still reproducible, stop-and-go propagation of domain walls, where the domain walls become statically pinned (zero domain wall velocity) at certain positions along the ring. Between *f*=3.75 and 5 MHz (green), the domain wall velocity periodically oscillates because of the intrinsic domain wall spin-structure oscillations. Above 10 MHz (blue), we observe domain wall motion above the Walker breakdown, in which the domain wall spin structure undergoes periodic transformations into a transverse domain wall. The oscillation frequency is approximately constant for *f*=3.75–5 MHz (below the Walker breakdown) and continuously increases for higher frequencies *f*=10–17.5 MHz (above the Walker breakdown). These oscillations occur independently of the field amplitude, as shown in Fig. 5b for a constant field rotation frequency of 5 MHz, and for the field amplitudes that are strong enough to depin the vortex domain walls and that are smaller than the coercive field of the permalloy ring structures^24^. However, we observe a small but clearly identifiable increase in the maximum domain wall velocity for the higher field amplitude.

Our observations indicate that these periodic oscillations of the vortex domain wall spin structure occur due to the curved geometries of the nanowires in combination with a rotating magnetic field and are directly correlated with oscillations of the domain wall velocity. We see that for vortex domain walls, these oscillations can occur even without transformation into a transverse domain wall and thus below the Walker breakdown. Further, for faster field rotation we observe domain wall motion above the Walker breakdown and this also leads to an oscillatory wall velocity directly confirming previous predictions^2^. Thus the oscillations of the domain wall velocity occur below *and* above the Walker breakdown and present a general feature in application-relevant curved geometries^19^,^20^.

### Intrinsic and extrinsic domain wall velocity variations

For sufficiently slow field-rotation frequencies, as shown in Fig. 5a for 2.5 MHz (red), we observe pinning-dominated domain wall propagation because of extrinsic effects, such as defects and imperfections in the nanowire. These are expected to give rise to a spatially varying energy landscape for domain walls^3^, and the interaction of the moving domain walls with the local pinning potential is expected to result in velocity variations due to extrinsic pinning. In particular, it has been predicted that these variations depend on the wall velocity (kinetic pinning^17^).

**Figure 6:**
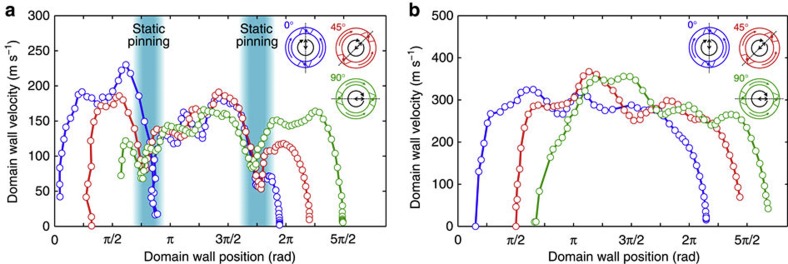
Intrinsic and extrinsic domain wall velocity variations. The domain wall velocity is plotted as a function of the domain wall azimuthal coordinate, recorded for a full field rotation at (**a**) *f*=7 MHz and (**b**) *f*=15 MHz, with different start and stop angles 0° (blue circles), 45° (red circles) and 90° (green circles), and constant field amplitude of *B*=6.8 mT. (**a**) We observed two static pinning events coinciding in all three velocity curves at around 0.75π rad and 1.75π rad. Furthermore, between π rad and 3π2 rad the domain wall velocities are strongly influenced by the local pinning potential landscape at non zero velocity (pinning dominated domain wall motion). The error of the domain wall velocity is *δv*_dw_=±25 m s^−1^. (**b**) The domain wall velocity oscillates and no indication of extrinsic pinning can be observed. The error of the domain wall velocity is *δv*_dw_=±52 m s^−1^.

To test this theory and to directly identify the extrinsic velocity variations at low wall velocities because of the local pinning potential landscape, we recorded the domain wall velocity for a complete field rotation with different start angles of 0° (blue), 45° (red) and 90° (green), as shown in Fig. 6. The field rotation frequencies are 7 MHz and 15 MHz, the field amplitude is *B*=6.8 mT and the ring structure is 500 nm wide and has a radius of 2.5 μm. For *f*=7 MHz (see Fig. 6a), we observe pinning-dominated domain wall propagation at an average domain wall velocity of 110 ms^−1^. The domain wall velocities are plotted as functions of the domain wall azimuthal coordinate. We observe two static pinning events at approximately 0.75πrad and 1.75π rad, at which the domain wall velocity drops close to zero. On the other hand, for *f*=15 MHz the domain wall velocity oscillates because of the intrinsic periodic domain wall spin structure changes, and there is no indication of extrinsic pinning, see Fig. 6b. This allows us to directly confirm the theory predictions of static and kinetic pinning. Moreover, by direct imaging of the domain propagation, we also directly probe the interplay between moving domain walls and the local pinning potential, revealing intrinsic and extrinsic domain wall velocity variations. Note that the field-rotation frequency that separates the pinning-dominated domain wall propagation regime from freely oscillating domain wall motion strongly depends on the depth of the pinning potential landscape due to defects and imperfections in the nanowire, and hence, on the sample under investigation.

In contrast to recent studies^3^,^5^,^6^, where limited control of the domain wall propagation due to stochasticity (which we also observe for slow domain walls) arising from the pinning potential landscape has been reported, we can here obtain very reliable domain wall propagation at faster field rotation frequencies that is fully reproducible billons of times. This control of the domain wall propagation is achieved by tailoring the driving field pulses to take advantage of the intrinsic wall spin-structure oscillations by driving the wall fast enough to overcome extrinsic pinning and by driving the system at timescales that are much shorter than thermally activated domain wall motion^30^.

## Discussion

Our surprising observation of oscillatory domain wall spin-structure changes during propagation below the Walker breakdown has not been previously observed and has not even been predicted. We explain the underlying mechanisms of this behaviour by considering the forces that act on the vortex domain wall spin structure, as schematically illustrated in Fig. 2b. The tangential field component *B*_t_=*B*sin*θ* acts both as an azimuthal driving force for the domain wall motion (not shown) and as a radial force **F**_t_ on the vortex core^2^:


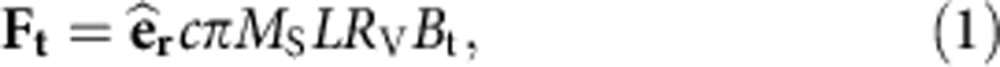


where *M*_S_ is the saturation magnetization, *c* is the chirality of the vortex domain wall and *L*, *R*_V_ are the thickness and radius of the vortex structure, respectively. In addition, the gyroforce **G** × **v**, with the gyrovector **G**^31^,^32^, points parallel to **F**_t_ for *p*=+1 and CCW rotating magnetic fields. Finally, a third force that acts on the vortex core is the restoring force from the parabolic potential energy due to the shape anisotropy of the ring structure, which only depends on the radial vortex core position. The interplay between the domain wall spin structure and these forces leads to oscillating domain wall propagation below the Walker breakdown, as shown in Figs 2a,b and 4a: At *t*=96ns, the gyroforce reaches the maximum at the highest domain wall velocity, pushing the vortex core towards the outer edge of the ring, while the force **F**_t_ decreases with the shrinking angle *θ*, because the domain wall is moving faster than the driving field. At *t*=112ns, the domain wall has overtaken the rotating field and therefore the driving force decelerates the domain wall. The force **F**_t_ points towards the ring center and the gyroforce is decreasing, such that the vortex core moves towards the center of the nanowire. At *t*=128 ns, the domain wall velocity is lowest and is smaller than the angular velocity of the rotating magnetic field, hence the force **F**_t_ increases, pushing the vortex core towards the outer edge of the nanowire. When the phase difference is largest between *t*=128–144ns, both the driving force and **F**_t_ are maximal. Hence the domain wall is accelerating and the gyroforce increases, pushing the vortex core toward the outer edge of the nanowire. It is this interaction of the vortex domain wall spin structure with the rotating magnetic field that leads to intrinsically oscillating domain wall propagation in curved nanowires.

**Figure 7:**
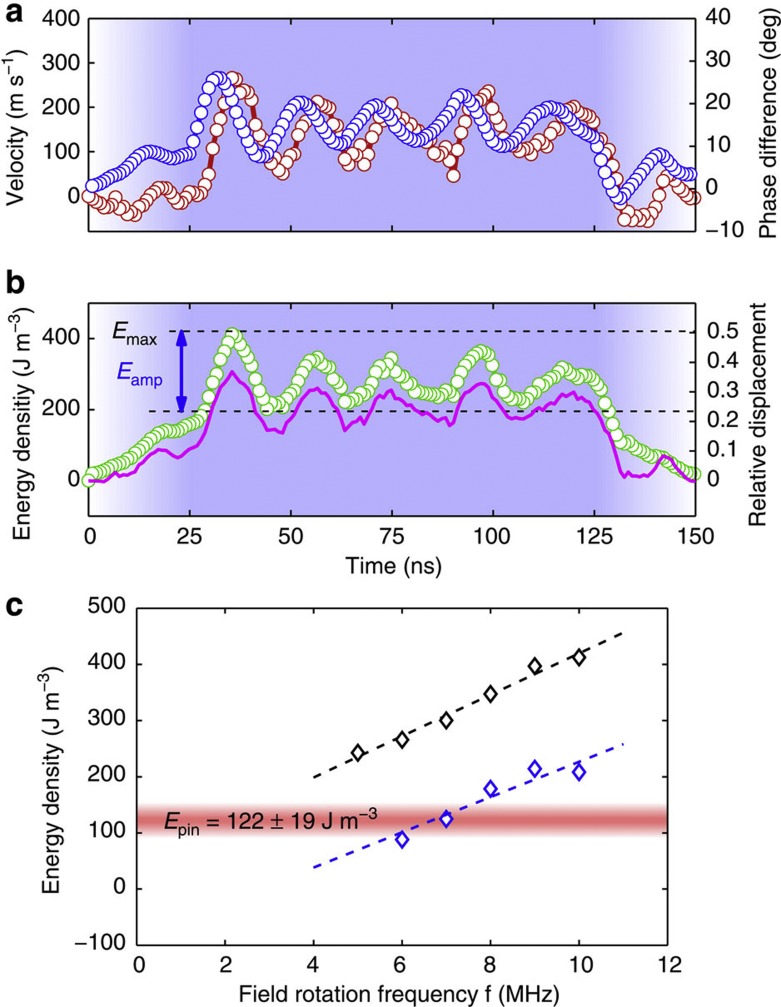
Enhanced magnetostatic domain wall energy. (**a**) Domain wall velocity *v*_dw_ (red circles) and the phase difference *θ* (blue circles) are plotted as functions of time, as obtained from micromagnetic simulations. The field rotation frequency is 10 MHz and the field amplitude is *B*=5 mT. The field amplitude of the rotating magnetic field is indicated by the saturation of the background color. (**b**) The normalized magnetostatic energy density (green circles) and the relative vortex core displacement (magenta line) plotted as functions of time. The magnetostatic energy is normalized to the energy of the remanent magnetization configuration, and the relative vortex core displacement corresponds to the distance from centre to the outer ring edge normalized to the ring width. (**c**) The maximum *E*_max_ (black diamonds) and the amplitude *E*_amp_ (blue diamonds) of the normalized magnetostatic energy density are plotted as functions of the field rotation frequency. The dotted lines show the linear interpolation of *E*_max_ and *E*_amp_, respectively. The experimentally measured average Zeeman depinning energy *E*_pin_=122±19 J m^−3^ is indicated by the horizontal red bar.

Such an interaction between the radial vortex core displacement and the domain wall velocity is not present in the analytical description of the one-dimensional model (1D)^2^,^33^, showing that the oscillating domain wall propagation in curved wires below and above the Walker breakdown goes beyond the simple 1D framework (for details, see Supplementary Note 1). This further highlights that in our case the walls cannot be described by point-like quasiparticles as in this simple model, but rather the full micromagnetic spin structure needs to be taken into account to explain the complex behaviour. To understand the reasons for the changes in the domain wall spin structure that go beyond the simple 1D framework quantitatively, we performed micromagnetic simulations^34^ (see Methods). The ring structure simulated is 750 nm wide, 30 nm thick and has a radius of 2 μm, and the field rotation frequency is *f*=10 MHz and the field strength used is *B*=5 mT. The results are shown in Fig. 7a and are found to be in good qualitative agreement with the experimental results. The timescale is faster because of the higher field rotation frequency to shorten the simulation time and to reduce edge roughness effects due to cell discretization. The oscillations of the domain wall velocity and the evolution of the phase difference are well reproduced in the simulations and coincide with changes in the vortex domain wall spin structure, with the radial oscillating vortex core position. As expected, the phase difference *θ* is larger for 10 MHz field rotation frequency in the simulations compared with the experiment.

To reveal the underlying energetics, the correlation of the normalized magnetostatic energy density and the radial vortex core position is shown in Fig. 7b. The normalized magnetostatic energy density oscillates with the relative vortex core displacement, because the magnetostatic energy of the domain wall can be described by a parabolic potential (see Supplementary Note 2). It is the gyroforce **G** × **v** and the force **F**_t_∝sin*θ* that push the vortex core radially and increase the potential energy. Both the gyroforce and **F**_t_ are, on average, proportional to the field rotation frequency because both the *v*_dw_ and *θ* depend linearly on *f* (below the Walker breakdown). Therefore, the maximum amplitude of the magnetostatic energy oscillations is higher for faster field-rotation frequencies. To show this, the oscillation amplitude *E*_amp_ and the energy maximum *E*_max_ of the magnetostatic energy (as defined in Fig. 7b) are plotted for various field-rotation frequencies between 5 and 10 MHz. As expected, *E*_amp_ and *E*_max_ increase approximately linearly. The magnetostatic energy is the main contribution to the internal domain wall energy of the vortex domain wall because the anisotropy energy is zero and the exchange energy concentrated in the vortex core is small and constant. Therefore, the internal domain wall energy periodically oscillates and the maximum scales linearly with the field rotation frequency and hence with the vortex domain wall velocity.

We conclude that the magnetostatic energy can act as an energy reservoir, leading to domain wall inertia and that the stored energy allows the domain wall to overcome a local pinning potential. To complete the picture, we estimate the depinning energy *E*_pin_ by the Zeeman energy density associated with the static depinning field, which is the field necessary to depin a statically pinned domain wall. As we observe reproducible pinning-dominated stop-and-go propagation of domain walls at 2.5 MHz field rotation frequency (see Fig. 5a), we can approximate the depinning field with the highest tangential field *B*_t,max_=*B*sin*θ*_max_ when the phase difference between the rotating field and the domain walls is largest. The Zeeman energy density of the remanent domain wall configuration, at an angle of *θ*_max_ with an external magnetic field of *B*=6.8 mT, gives our estimate for the depinning energy density *E*_pin_=122±19 J m^−3^, as indicated in Figure 7c by the horizontal red bar. In the micromagnetic simulations the amplitude of the magnetostatic oscillations *E*_amp_ is higher than the depinning energy density *E*_pin_ for field rotation frequencies above 7 MHz, providing a quantitative estimate of the domain wall velocity separating the extrinsic pinning-dominated domain wall propagation regime and the freely and intrinsically oscillating domain wall propagation regime below and above the Walker breakdown.

In conclusion, we have directly imaged oscillating vortex domain wall propagation below and above the Walker breakdown in curved nanowires driven by rotating magnetic fields. These oscillations of the vortex domain wall velocity are consequences of the interplay between the rotating driving field and the periodically changing domain wall spin structure. The relevant forces are proportional to the field rotation frequency and therefore the magnetostatic energy potential scales with the domain wall velocity, making fast domain walls less susceptible to pinning. This explains our observations of pinning of domain walls at defects at low wall velocities, whereas at high velocities the walls can overcome pinning. This pump and probe experiment demonstrates precise control of the domain wall position at high domain wall velocity, enabling a reliable and reproducible high-speed domain wall manipulation in magnetic logic and sensing devices.

## Methods

### Experiment

The permalloy Ni_80_Fe_20_(30 nm)/Au(2 nm) ring structures were fabricated on top of a 100 nm thick silicon nitride membrane, and Cr(4 nm)/Cu(150 nm) striplines are fabricated on top of the ring structures. All structures were prepared by electron-beam lithography and lift-off processing. The permalloy was deposited by molecular beam evaporation in UHV and the copper was thermally evaporated. The in-plane rotating magnetic fields were generated using sinusoidal current burst pulses, injected with a 90° phase shift in orthogonal striplines^29^, see inset Fig. 1. The field is homogeneous in the centre of the striplines and the error of the field amplitude is less than ±10% within the area occupied by the ring structures. The in-plane component of the magnetization was imaged using time-resolved scanning transmission X-ray microscopy, where the sample is tilted by 60° with respect to the X-ray beam and by taking advantage of the X-ray magnetic circular dichroism^35^. The data are recorded at the Ni L_3_ absorbtion edge (852.7 eV). The lateral resolution is ≈30 nm and the temporal resolution is dominated by the jitter of the electronics ≈250 ps. The repetition rate of the stroboscopic experiment is between 425 and 832 kHz and the transmission signal is recorded over >10^9^ subsequent pulsecycles. Snapshots of the dynamic magnetic contrast are then taken with a Δ*t*=2 ns time interval. The vortex core positions ***r***_*n*_ are determined for each image from the black–white contrast manually. The instantaneous domain wall velocity is then calculated by applying a moving average filter


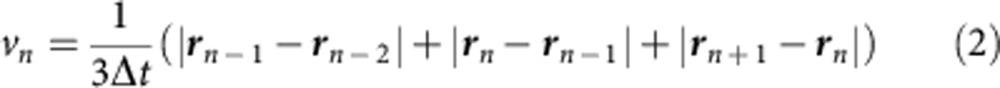


and the error is the standard deviation of the differences between the filtered and non-filtered velocities


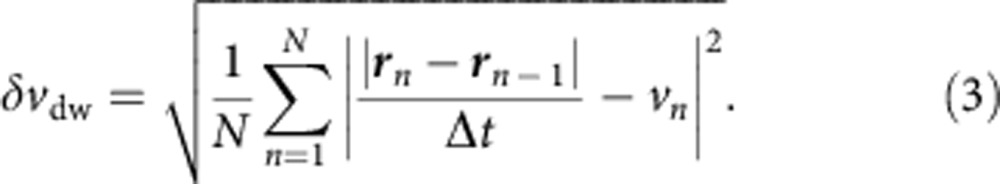


### Micromagnetic simulations

The micromagnetic simulations were carried out with the object-oriented micromagnetic framework^34^. The 750 nm wide and 30 nm thick ring structure with 2 μm diameter was simulated with a cell size of 5 × 5 × 30 nm^3^, assuming typical material parameters for permalloy^36^: *M*_S_=800 kA m^−1^, exchange stiffness *A*=13 × 10^12^ J m^−1^, damping parameter *α*=0.008 and anisotropy constant *K*_1_=0. The configuration of the vortex domain walls (*p*=+1, *c*=±1) and the CCW sense of rotation of the rotating magnetic field burst pulses are chosen as in the experiment; the field rotation frequency is *f*=5–10 MHz. The position of the vortex core was determined by fitting a Gaussian through the *M*_*z*_ component of the magnetization.

## Author contributions

A.B., M.S., M.-A.M., J.R. and M.K. conceived the project; A.B., M.-A.M. and J.H. prepared the samples; A.B., M.S., M.-A.M., C.M., F.B., M.N., M.W., T.T. and M.K. carried out the measurements; micromagnetic simulations and data analysis are performed by A.B. and M.S.; A.B., M.-A.M., M.K. and F.B. prepared the manuscript; S.E., B.V.W., H.S., G.S. and M.K. supervised the project. All authors discussed the results.

## Additional information

**How to cite this article:** Bisig, A. *et al.* Correlation between spin structure oscillations and domain wall velocities. *Nat. Commun.* 4:2328 doi: 10.1038/ncomms3328 (2013).

## Supplementary Material

Supplementary Figures, Notes and References Supplementary Figures S1-S2, Supplementary Notes 1-2 and Supplementary References

Supplementary Movie 1 Time-resolved movie of oscillating domain wall propagation. A series of time resolved scanning transmission X-ray microscopy snapshots with 2 ns time resolution of moving domain walls during a 5 MHz rotational field burst pulse is shown. Black (white) contrast indicates the magnetization pointing to the left (right). The direction and the strength of the rotating magnetic field is indicated by the green arrow and the vortex positions are indicated by the red dots.

## References

[b1] Schryer N. L. & Walker L. R. The motion of 180° domain walls in uniform dc magnetic fields. J. Appl. Phys. 45, 5406–5421 (1974).

[b2] Thiaville A. & Nakatani Y. Domain-wall dynamics in nanowires and nanostrips. In (eds.) Hillebrands B., Thiaville A. Spin Dynamics in Confined Magnetic Structures III, vol. 101 of Topics in Applied Physics 161–205Springer (2006).

[b3] Jiang X. *et al.* Enhanced stochasticity of domain wall motion in magnetic racetracks due to dynamic pinning. Nat. Commun. 1, 25 (2010). 20975690 10.1038/ncomms1024

[b4] Hayashi M., Thomas L., Rettner C., Moriya R. & Parkin S. S. P. Direct observation of the coherent precession of magnetic domain walls propagating along permalloy nanowires. Nat. Phys. 3, 21–25 (2007).

[b5] Briones J. *et al.* Stochastic and complex depinning dynamics of magnetic domain walls. Phys. Rev. B 83, 060401 (2011).

[b6] Jiang X., Thomas L., Moriya R. & Parkin S. S. P. Discrete domain wall positioning due to pinning in current driven motion along nanowires. Nano. Lett. 11, 96–100 (2011). 21162554 10.1021/nl102890h

[b7] Rhensius J. *et al.* Imaging of domain wall inertia in permalloy half-ring nanowires by time-resolved photoemission electron microscopy. Phys. Rev. Lett. 104, 067201 (2010). 20366851 10.1103/PhysRevLett.104.067201

[b8] Thomas L., Moriya R., Rettner C. & Parkin S. S. P. Dynamics of magnetic domain walls under their own inertia. Science 330, 1810–1813 (2010). 21205666 10.1126/science.1197468

[b9] Döring W. Über die Trägheit der Wände zwischen Weißchen Bezirken. Z. Naturforsch 3a, 373–379 (1948).

[b10] Rado G. T. On the inertia of oscillating ferromagnetic domain walls. Phys. Rev. 83, 821–826 (1951).

[b11] Saitoh E., Miyajima H., Yamaoka T. & Tatara G. Current-induced resonance and mass determination of a single magnetic domain wall. Nature 432, 203–206 (2004). 15538364 10.1038/nature03009

[b12] Bedau D. *et al.* Detection of current-induced resonance of geometrically confined domain walls. Phys. Rev. Lett. 99, 146601 (2007). 17930695 10.1103/PhysRevLett.99.146601

[b13] Thomas L. *et al.* Resonant amplification of magnetic domain wall motion by a train of current pulses. Science 315, 1553–1556 (2007). 17363668 10.1126/science.1137662

[b14] Bocklage L. *et al.* Time-resolved imaging of current-induced domain wall oscillations. Phys. Rev. B 78, 180405(R) (2008).

[b15] Chauleau J.-Y., Weil R., Thiaville A. & Miltat J. Magnetic domain walls displacement: automotion versus spin-transfer torque. Phys. Rev. B 82, 214414 (2010).

[b16] Ahn S.-M., Moon K.-W., Kim D.-H. & Choe S.-B. Detection of the static and kinetic pinning of domain walls in ferromagnetic nanowires. Appl. Phys. Lett. 95, 152506 (2009).

[b17] Ahn S.-M., Kim D.-H. & Choe S.-B. Kinetic and static domain wall pinning at notches on ferromagnetic nanowires. IEEE Trans. Magn. 45, 2478–2480 (2009).

[b18] Lewis E. R. *et al.* Kinetic depinning of a magnetic domain wall above the walker field. Appl. Phys. Lett. 98, 042502 (2011).

[b19] Allwood D. A. *et al.* Magnetic domain wall logic. Science 309, 1688–1692 (2005). 16151002 10.1126/science.1108813

[b20] Diegel M., Mattheis R. & Halder E. Multiturn counter using movement and storage of 180° magnetic domain walls. Sens. Lett. 5, 118–122 (2007).

[b21] McMichael R. & Donahue M. Head to head domain wall structures in thin magnetic strips. IEEE Trans. Magn. 33, 4167–4169 (1997).

[b22] Laufenberg M. *et al.* Observation of thermally activated domain wall transformations. Appl. Phys. Lett. 88, 052507 (2006).

[b23] Rothman J. *et al.* Observation of a bi-domain state and nucleation free switching in mesoscopic ring magnets. Phys. Rev. Lett. 86, 1098–1101 (2001). 11178019 10.1103/PhysRevLett.86.1098

[b24] Kläui M., Vaz C. A. F., Lopez-Diaz L. & Bland J. A. C. Vortex formation in narrow ferromagnetic rings. J. Phys. Condens. Matter 15, R985–R1024 (2003).

[b25] Kläui M. Head-to-head domain walls in magnetic nanostructures. J. Phys. Condens. Mattter 20, 313001 (2008).

[b26] Negoita M., Hayward T. J. & Allwood D. A. Controlling domain walls velocities in ferromagnetic ring-shaped nanowires. Appl. Phys. Lett. 100, 072405 (2012).

[b27] Kilcoyne A. L. D. *et al.* Interferometer-controlled scanning transmission x-ray microscopes at the advanced light source. J. Synchrotron Radiat. 10, 125–136 (2003). 12606790 10.1107/s0909049502017739

[b28] Curcic M. *et al.* Polarization selective magnetic vortex dynamics and core reversal in rotating magnetic fields. Phys. Rev. Lett. 101, 197204 (2008). 19113302 10.1103/PhysRevLett.101.197204

[b29] Curcic M. *et al.* Magnetic vortex core reversal by rotating magnetic fields generated on micrometer length scales. Phys. Status Solidi. B 248, 2317–2322 (2011).

[b30] Eltschka M. *et al.* Nonadiabatic spin torque investigated using thermally activated magnetic domain wall dynamics. Phys. Rev. Lett. 105, 056601 (2010). 20867942 10.1103/PhysRevLett.105.056601

[b31] Thiele A. A. Steady-state motion of magnetic domains. Phys. Rev. Lett. 30, 230–233 (1973).

[b32] Huber D. L. Dynamics of spin vortices in two-dimensional planar magnets. Phys. Rev. B 26, 3758–3765 (1982).

[b33] Malozemoff A. P. & Slonczewski J. C. Magnetic domain walls in bubble material. Academic: New York, (1979).

[b34] Donahue M. & Porter D. Oommf User’s Guide, Version 1.0. Interagency Report NISTIR 6376 National Institute of Standards and Technology (1999).

[b35] Schütz G. *et al.* Absorption of circularly polarized x rays in iron. Phys. Rev. Lett. 58, 737–740 (1987). 10035022 10.1103/PhysRevLett.58.737

[b36] Moore T. A. *et al.* Scaling of spin relaxation and angular momentum dissipation in permalloy nanowires. Phys. Rev. B 80, 132403 (2009).

