# Supplementary material for: Correlation between spin structure oscillations and domain wall velocities
Source: Nat Commun. 2013 Aug 27;4:2328. doi: 10.1038/ncomms3328 (PMC3759078; doi:10.1038/ncomms3328)
Supplement: Supplementary Figures, Notes and References — Supplementary Figures S1-S2, Supplementary Notes 1-2 and Supplementary References [file ncomms3328-s1.pdf]

Supplementary Figure S1

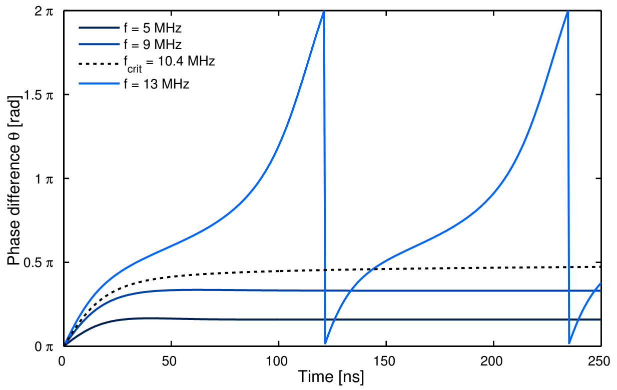

1D domain wall model for circular nanowires and rotating magnetic fields. The phase difference θ is plotted as a function of the time for different field rotation frequencies $f = 5{-}13$ MHz. The field strength is $B = 0.3$ mT, below the Walker field $\mu_0 H_{\mathrm{W}} = 0.4$ mT and the critical field rotation frequency in this case is $f_{\mathrm{crit}} = 10.4$ MHz. The steady-state motion for $f < f_{\mathrm{crit}}$ is at constant velocity without precession of the polar angle ψ and the phase difference θ is constant. For $f > f_{\mathrm{crit}}$, the domain wall cannot track the rotating magnetic field and θ grows continuously.

Supplementary Figure S2

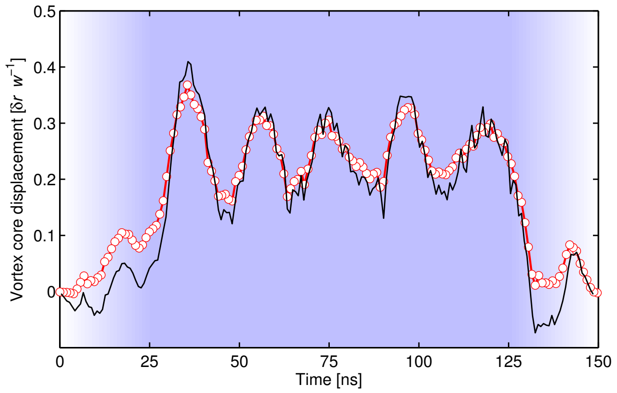

Radial vortex core displacement. The calculated vortex core displacement δr (black line) and the radial vortex core position from micromagnetic simulations (red circles) are plotted as a function of time. By fitting the radial potential stiffness κ_r we find very good quantitative agreement between the analytical expression for δr and the vortex core position in the micromagentic simulation.

Supplementary Note 1

Domain wall motion can be analytically described by a one-dimensional (1D) model for the collective coordinates domain wall position q and polar angle ψ [34]. Here we derive the 1D model for domain wall motion in circular nanowires driven by a rotating magnetic field and we numerically find that in contrast to our observation the steady-state domain wall motion below the Walker breakdown occurs at constant velocity and without precession (constant polar angle ψ, corresponding to no changes in the domain wall spin structure).
The driving magnetic field rotates at constant angular velocity and constant amplitude

$$\mathbf{B} = B \cdot [\sin(2\pi f t), \cos(2\pi f t)]\,. \tag{S1}$$

To reflect the circular geometry, we assume periodic boundary conditions for the domain wall position q

$$(q, t) \equiv (q + n\pi 2R, t), \qquad n \in \mathbb{Z}, \tag{S2}$$

where R is the radius of the circular nanowire. The driving field is the tangential component $B_\mathrm{t} = B\sin(\theta)$ of the rotating field and θ is the angle between the magnetic field and the azimuthal position of the domain wall. Neglecting the pinning potential, we can write the 1D model for domain wall motion in circular nanowires and rotating magnetic fields:

$$\begin{aligned} \Delta_0\dot{\psi} - \alpha\dot{q} &= \gamma_0\Delta_0 B_\mathrm{t} \\ \dot{q} + \alpha\Delta_0\dot{\psi} &= -\frac{\gamma_0\Delta_0}{M_\mathrm{S}} K_\mathrm{d}\sin(2\psi), \end{aligned} \tag{S3}$$

where γ_0 is the gyromagnetic ratio, α is the Gilbert damping parameter, M_S is the saturation magnetization, K_d is the transverse anisotropy and Δ_0 is the domain wall width in equilibrium.
To numerically solve the model, we assume realistic material parameters and dimensions of the ring structure [37, 38]: $M_\mathrm{S} = 800 \times 10^3$ A m^{-1}, $\alpha = 0.008$, $K_\mathrm{d} = 4 \times 10^4$ J m^{-3}, $\Delta_0 = 20$ nm and $r = 2\mu$m. The initial conditions of the equations of motion are $q_0 = 0$ and $\psi_0 = 0$ and the angle of the rotating magnetic field coincides with the domain wall position ($\theta_0 = 0$).
We consider only the case where B_t is smaller than the Walker field $\mu_0 H_\mathrm{W} = \alpha K_\mathrm{d}/M_\mathrm{S}$, where the domain wall velocity is proportional to the tangential field amplitude [2]

$$v_\mathrm{dw} = \gamma_0\Delta_0\frac{B_\mathrm{t}}{\alpha}. \tag{S4}$$

The maximum domain wall velocity at $B_\mathrm{t} = B$ ($\theta = \pi/2$) defines a critical field rotation frequency for the domain wall to follow the field

$$f_\mathrm{crit} = \frac{\gamma_0 \Delta_0 B}{2\pi\alpha R}. \tag{S5}$$

For $f < f_\mathrm{crit}$, the domain wall lags behind the rotating field ($\theta > 0$) and the tangential field component grows until the domain wall velocity matches the field rotation speed ($v_\mathrm{dw} = 2\pi f R$). The steady-state motion in this case is at constant velocity without precession of the polar angle ψ, as shown in Supplementary Figure S1. If the field rotation frequency exceeds the critical field rotation frequency $f > f_\mathrm{crit}$, the domain wall cannot track the the rotating magnetic field.

Supplementary Note 2

Here, we calculate analytically the radial vortex core displacement δr based on the velocities obtained from micromagnetic simulations. We assume a parabolic potential along the radial direction, due to the shape anisotropy:

$$V_r\left(\delta r\right) = \begin{cases} \frac{\kappa_r}{2}\delta r^2, & \delta r^2 < \left(\frac{w}{2}\right)^2, \\ V_0, & \delta r^2 \geq \left(\frac{w}{2}\right)^2, \end{cases} \tag{S6}$$

where w is the ring width, $V_0 = \kappa_r w^2/8$ is the potential depth and κ_r is the radial potential stiffness. Assuming that the restoring force $\mathbf{F}_\mathrm{rf} = -\hat{\mathbf{e}}_\mathbf{r}\partial V_r/\partial\delta r$ from the parabolic potential counteracts the sum of the gyroforce $\mathbf{G} \times \mathbf{v}$ and $\mathbf{F}_\mathrm{t}$, we can calculate the radial vortex core displacement δr

$$\delta r = \frac{|\mathbf{G} \times \mathbf{v} + \mathbf{F}_\mathrm{t}|}{\kappa_r}, \tag{S7}$$

because the gyroforce and $\mathbf{F}_\mathrm{t}$ are always pointing parallel. The potential stiffness κ_r is found by fitting δr to the vortex core displacement from the micromagnetic simulations, the best fit is found for $\kappa_r = 8.9 \pm 0.1 \times 10^{-4}$ kg s^{-2}, which agrees quantitatively with previous measurements [39]. The resulting time evolution of the vortex core displacement and the radial vortex core position from the micromagnetic simulations are shown in Supplementary Figure S2.

Supplementary References

[38] Yang, J., Nistor, C., Beach, G. S. D. & Erskine, J. L. Magnetic domain-wall velocity oscillations in permalloy nanowires. *Phys. Rev. B* **77**, 014413 (2008).

[39] Bisig, A. *et al.* Direct imaging of current induced magnetic vortex gyration in an asymmetric potential well. *Appl. Phys. Lett.* **96**, 152506 (2010).
